# The strategic applications of natural polymer nanocomposites in food packaging and agriculture: Chances, challenges, and consumers’ perception

**DOI:** 10.3389/fchem.2022.1106230

**Published:** 2023-01-10

**Authors:** Magdalena Wypij, Joanna Trzcińska-Wencel, Patrycja Golińska, Graciela Dolores Avila-Quezada, Avinash P. Ingle, Mahendra Rai

**Affiliations:** ^1^ Faculty of Biological and Veterinary Sciences, Nicolaus Copernicus University, Toruń, Poland; ^2^ Facultad de Ciencias Agrotecnologicas, Universidad Autonoma de Chihuahua, Chihuahua, Mexico; ^3^ Department of Agricultural Botany, Biotechnology Centre, Dr. Panjabrao Deshmukh Krishi Vidyapeeth, Akola, India; ^4^ Nanobiotechnology Laboratory, Department of Biotechnology, Sant Gadge Baba Amravati University, Amravati, India

**Keywords:** agriculture, biopolymer, crop improvement, food packaging, nanocarriers, nano-coating, nanocomposite, slowrelease hydrogel

## Abstract

Natural polymer-based nanocomposites have received significant attention in both scientific and industrial research in recent years. They can help to eliminate the consequences of application of petroleum-derived polymeric materials and related environmental concerns. Such nanocomposites consist of natural biopolymers (e.g., chitosan, starch, cellulose, alginate and many more) derived from plants, microbes and animals that are abundantly available in nature, biodegradable and thus eco-friendly, and can be used for developing nanocomposites for agriculture and food industry applications. Biopolymer-based nanocomposites can act as slow-release nanocarriers for delivering agrochemicals (fertilizers/nutrients) or pesticides to crop plants to increase yields. Similarly, biopolymer-based nanofilms or hydrogels may be used as direct product coating to extend product shelf life or improve seed germination or protection from pathogens and pests. Biopolymers have huge potential in food-packaging. However, their packaging properties, such as mechanical strength or gas, water or microbial barriers can be remarkably improved when combined with nanofillers such as nanoparticles. This article provides an overview of the strategic applications of natural polymer nanocomposites in food and agriculture as nanocarriers of active compounds, polymer-based hydrogels, nanocoatings and nanofilms. However, the risk, challenges, chances, and consumers’ perceptions of nanotechnology applications in agriculture and food production and packaging have been also discussed.

## 1 Introduction

The world population is estimated to be eight billion in 2024 and 9.7 billion by 2050 (Godfray, 2014; Lahlali et al., 2022). According to another estimation globally, 33% of the food produced is deteriorated or wasted (Motelica et al., 2020). The pathogens and pests are responsible for the deterioration of food crops, among others. This loss can be saved by using protection methods. The surge in population and food deterioration will demand additional food supply, and therefore food security is an important issue. The traditional way of food protection is not enough, we need to search for alternative technologies such as nanotechnology.

In the present scenario, nanotechnology has revolutionized all the fields of life including medicine, agriculture, energy, electronics, etc. The basis of this technology is nanomaterials that are in the range of 1–100 nm (Rai et al., 2021a). Natural polymers also known as biopolymers are abundantly available in nature, and are biodegradable and thus eco-friendly (Ashfaq et al., 2019; Karimi Sani et al., 2023). These include but are not limited to starch, cellulose, chitosan, and alginate resulting from plants, animals or microbes including fungi, algae, and bacteria. Other forms of natural polymer are nucleic acids including DNA and RNA. Silk, wool, and honey are also natural polymers that are used in daily life (Ganesan, 2017; Chaudhary et al., 2020; Panchal and Vasava, 2022).

Biopolymers (e.g., chitosan, alginate, fibrin, and hyaluronic acid) play a remarkable role in the absorption of water (biopolymer-based hydrogels, super absorbent) in soil (Tomadoni et al., 2019) and can also be used as slow-releasing nanocarriers for the delivery of agrochemicals to plants (fertilizers/nutrients) and also for treating pathogens (fungicides, bactericides, virucides) and pests (pesticides) (Sikder et al., 2021). The release of agrochemicals is slowed down drastically as a consequence, and the leaching of the agrochemicals into the soil and aquatic ecosystem minimizes. The polymer-based hydrogels can be used as absorbing agents, slow release agents for agrochemicals and seed-coating materials for easy germination by keeping away seeds from pathogens and pests (Tomadoni et al., 2019).

Similarly, natural polymers such as proteins, polysaccharides, lipids, and their combinations or composites with nanoparticles (e.g., ZrO_2_) can be used as biodegradable and/or edible films in food packaging that can be alternatives to non-biodegradable packaging materials (Karimi Sani et al., 2023).

Although the use of natural polymers in e.g., food industry, food packaging, and agriculture is beneficial from eco-friendly point of view, there are some inherent limitations resulting from their properties, such as poor stability, mechanical strength, and rapid degradation, that need to be improved to produce a favorable product for these sectors. These challenges can be overcome when natural polymers are used as encapsulating agents for the nanoparticles such as AgNPs, AuNPs, CuNPs, SiNPs, MgNPs, etc. (Karimi Sani et al., 2021; Reddy et al., 2021; Taherimehr et al., 2021; Rofeal et al., 2022). It is hoped that natural polymers and their composites will significantly benefit food and agriculture by ensuring food security and sustainability (Sikder et al., 2021; Rofeal et al., 2022).

In this review, we have discussed the role of eco-friendly natural polymer nanocomposites in food and agriculture. In addition, we have analyzed the opportunities, challenges, and risks (toxicity) concerning the use of nanomaterials in food and agriculture and also the public perception of such applications that are essentially required for acceptance of new technology.

## 2 Polymer-nanocomposites

**FIGURE 1:**
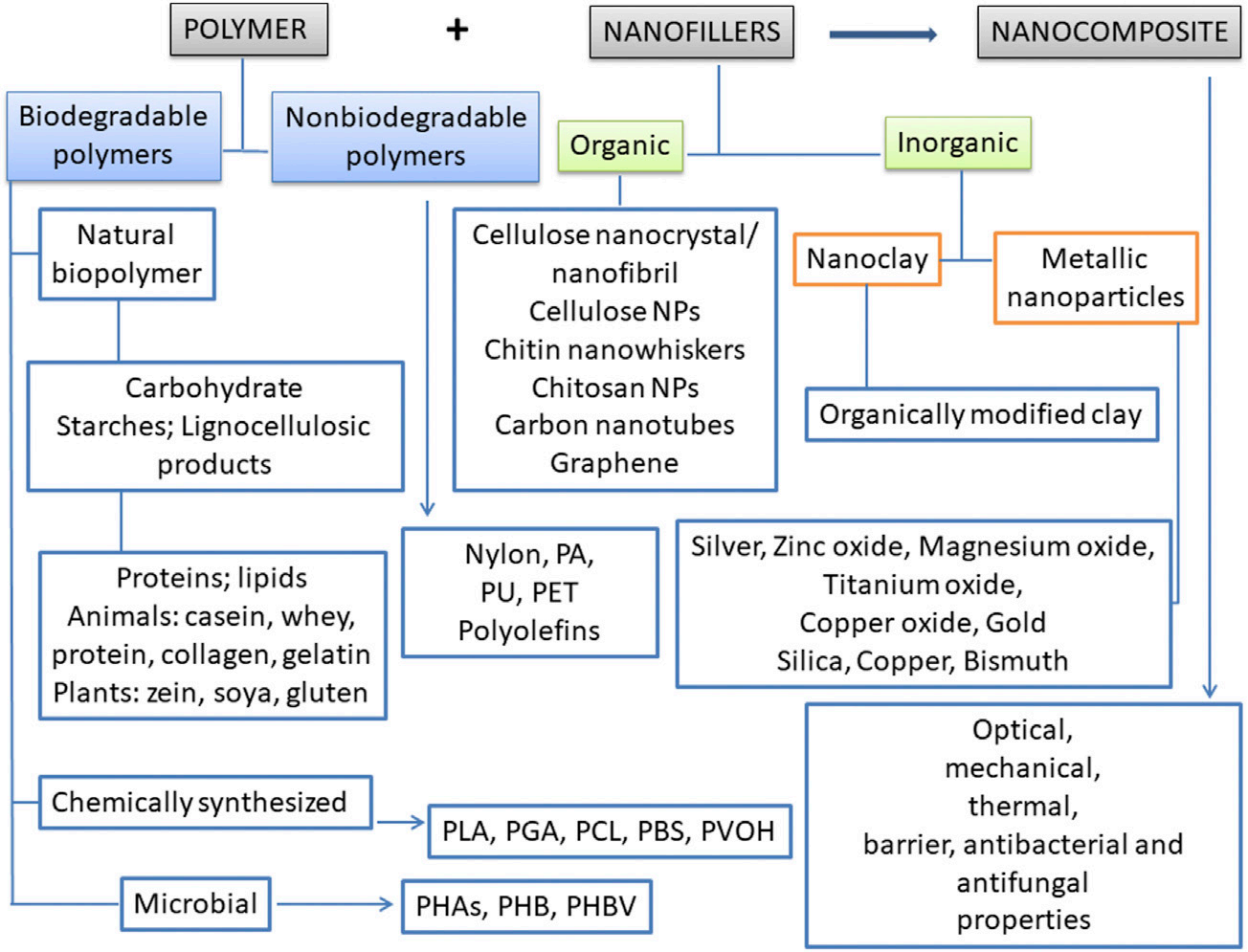
Polymer-nanocomposite structures. PA, Polyamide/Nylon; PBS, Polybutylene succinate; PCL, Polycaprolactone; PET, Polyethylene terephthalate; PGA, Polyglycolic Acid; PHAs, Polyhydroxyalkanoates; PHB, Polyhydroxybutyrate; PHBV, Poly (3-hydroxybutyrate-co-3-hydroxyvalerate); PLA, Polylactic acid; PU, Polyurethane; PVOH, Polyvinyl Alcohol.

The polymer nanocomposite is considered to multiphase hybrid solid material that contains one of the phases as nanoscale fillers that have at least one dimension in less than 100 nm distributed within a polymer matrix (Ray and Bousmina, 2005). Polymer nanocomposites are composed of the polymer matrix, nanofillers, plasticizers, and compatibilizers (Winey and Vaia 2007; Bustamante-Torres et al., 2021) (Figure 1).

The polymers used to preparation of nanocomposites could be classified as natural and chemically synthesized biodegradable polymers; microbial polyesters and also non-biodegradable polymers (Clarinval and Halleux, 2005; Bordes et al., 2009), as shown in Figure 1.

### 2.1 Polymer component of the nanocomposites

Biodegradable biopolymers are renewable resources of plant, animal, and microbiological origin (Pawar and Purwar, 2013) and obtained chemically with the use of natural starting materials such as fats or oils, sugars, and starch (Pawar and Purwar, 2013). These polymers can be degraded by microbes (Singh and Saini. 2014). The natural easy biodegradable polymeric materials include proteins (soy, wheat, corn, gluten, whey, collagen, gelatin, and albumin), polysaccharides (chitosan, alginates, starch, cellulose, and chitin), nucleic acids and lipids (bees, wax, and free fatty acids), carbohydrates (pullulan and curdlan), polyhydroxy butyrate (PHB), polyhydroxyalkanoic acids (Steinbüchel 2005; Mathew and Radhakrishnan, 2019; Chaudhary et al., 2020; Panchal and Vasava, 2022) while these slowly biodegradable compounds belong to polyphenols and polyisoprenoids that are represented by lignin and poly (cis-1,4-isoprene), respectively (Steinbüchel 2005).

**FIGURE 2:**
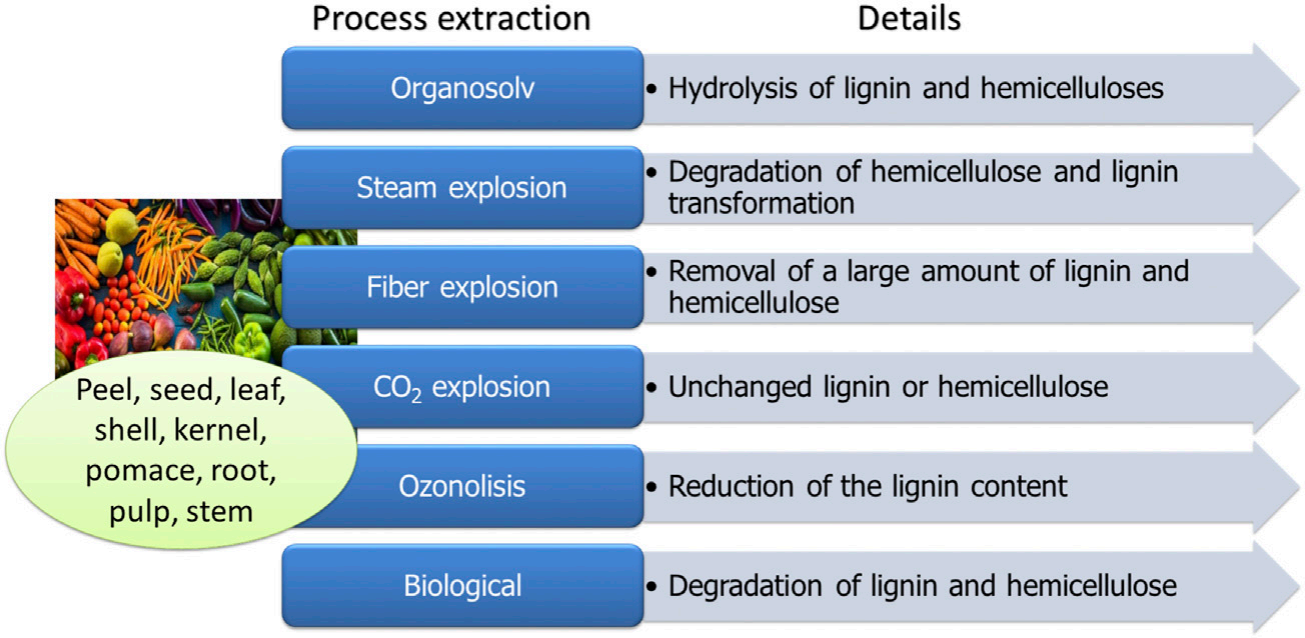
Illustrative processes for extracting substrates for biopolymer synthesis from food industry waste.

The cellulosic and hemicellulosic, starch, lignin which are abundantly available from waste-derived organic matter (e.g., wood, potatoes, maize, and wheat), are examples of low-cost substrates for the production of biopolymers. Extraction technologies (Figure 2) offer several advantages, such as cost efficiency, low energy requirements, and non-toxic waste, but there are some challenges, such as low efficiency and prolonged processing time (Jha and Kumar, 2019; Karimi Sani et al., 2023). For instance, the degradation of hemicellulose and transformation of lignin by the steam explosion is a low-cost process, but additional steps, such as ethanol extraction and purification, are required for complete biomass fractionation (Hongzhang and Liying, 2007).

The synthetic biodegradable biopolymers include poly (lactic acid) (PLA), poly-(glycolic acid) (PGA), poly (lactic-co-glycolic acid) (PLGA), poly (butylene succinate) (PBA), polycaprolactone (PCL), poly (ethylene adipate) (PEA), poly (p-dioxanone) (PDS), and their copolymers (Rodrigues and Vieira, 2019; Panchal and Vasava, 2022). In turn, non-biodegradable synthetic polymers can be categorized into high-density (HDPE) and low-density polyethylene (LDPE) including e.g., ethylene vinyl acetate (EVA), polyethylene terephthalate (PET), polyethylene (PE), polypropylene (PP) and polystyrene (PS) (Mathew and Radhskrishnan, 2019). In fact, non-degradable polymers have created several environmental problems and therefore biodegradable polymers are gaining ground (Clarinval and Halleux, 2005; Karimi Sani et al., 2023). For instance, the recently debated topic of micro- and nano-plastics which are responsible for adverse effects on soil, microbes, plants, animals, and humans. They can enter into the food chain and cause deleterious effects on the ecosystem. Such plastics interact with potentially toxic elements and cause toxicity to the soil (Allouzi et al., 2021).

The biodegradability of bio-based polymers depends on chemical and crystal structures glass transition temperature, hydrophilicity/hydrophobicity, mechanical properties, melting point and molecular weight (Saini, 2017; Sani et al., 2019; Vinod et al., 2020). The natural origin polymers such as those from fruits and vegetables degrade faster than synthetic polymers under environmental conditions by microorganisms decomposition and enzyme activity, as exemplified by the study of Zhao et al. (2019) who found that, in soil, cellulosic films made from durian rind were more biodegradable than cellophane for 4 weeks. The higher molecular weight, crystallinity or melting temperature, the lower biodegradability of the polymer. Similarly, the branched-chain polymers degrade slower than non-branched ones (Saini, 2017; Ganapathy et al., 2019; Karimi Sani et al., 2023). Moreover, at temperatures above the glass transition temperature, the polymer is soft and rubbery and the polymer film becomes highly permeable to gases. In turn, low temperatures bring polymers glassy and hard, and their films are less permeable to gases (Pirsa et al., 2022; Karimi Sani et al., 2023).

It is worth noting that the use of biopolymers in food products require Generally Recognized as Safe (GRAS) status approved by the United States Food and Drug Administration (FDA) (Moradali and Rehm, 2020). The biomaterials used should be non-toxic, safe for long-term use, non-allergenic, endotoxin-free, and derived from non-pathogenic strains of microorganisms. Well-studied edible natural biopolymers include, for example, polysaccharides (chitosan and alginate), proteins (isolated from soy and whey, gelatin, zein), and carbohydrates (pullulan) (Kraśniewska et al., 2017; Wang et al., 2022).

### 2.2 Properties of biopolymer-based nanocomposites

A significantly improved alternative to conventionally used polymers is nanocomposites, i.e., biopolymers reinforced by nanofillers (Mathew and Radhakrishnan, 2019). Many organic and inorganic materials such as silica NPs, carbon nanotubes (CNTs), nanosheets, graphene, silver, copper, zinc, titanium dioxide, copper oxide, zinc oxide, zirconium oxide nanoparticles, cellulose nanofibers, starch nanocrystals, chitosan, and chitin whiskers, clay nanomaterials (montmorillonite, kaolinite, halloysite, saponite, hectorite, and laponite) find application as fillers for improving polymer properties (Albdiry and Yousif, 2019; Lin et al., 2019; Olivera et al., 2019; Karimi Sani et al., 2021).

Properties of biopolymer-based nanocomposites stem from their controlled design, ordered structure, and incorporation of multifunctional nanocomponents. The desired properties are determined by their intended application and are acquired by optimization of the synthesis process (Camargo et al., 2009; Karimi Sani et al., 2023). Basically, nanocomposite systems are made of polymer matrix and incorporated nanosized components, as mentioned earlier. Among nanofillers are those in the form of particles, crystals, rods, whiskers, fibres, tubes or nanogels, and nanoemulsions, which are encapsulated into or deposited on the surface of the polymeric matrix (Priyadarshi et al., 2021). Techniques employed for evaluating nanocomposite characteristics such as structure, surface properties and morphology, crystallinity, composition or stability include X-ray diffraction analysis, Fourier Transform Infrared (FTIR) spectroscopy, thermogravimetric analysis (TGA), atomic force (AFM) or electron microscopic observations (TEM/SEM), etc. (Vasile, 2018). For instance, analysis of the structures of the AgNPs and chitosan nanofibers nanocomposites synthesized *in situ* (BP/AgP) or *ex situ* (BP/AgNP) showed surface and internally deposited AgNPs, respectively. *In situ* synthesis resulted in formation of ultra-small AgNPs (5 nm) with much higher homogeneity in size and narrow size distribution without agglomeration of AgNPs compared to *ex situ*, as confirmed by XRD and small angle X-ray scattering (SAXS) techniques, and SEM observations (Zienkiewicz-Strzała and Deryło-Marczewska, 2020). Leonardi et al. (2021) synthesized nanocomposite (with size 309 ± 56 nm) by CuONPs encapsulation in a biopolymeric (chitosan and sodium alginate) shell to slowly release Cu. Results from TGA confirmed 30% metal contribution to the fabricated nanoformula, while FTIR analysis showed that incorporation of CuONPs was related to the presence of amine (–NH_2_) and hydroxyl (–OH) groups in the polyelectrolyte complexes (PEC) formed from alginate and chitosan. The results from ICP-OES analysis demonstrated that encapsulation of CuONPs with PEC allowed slow release of CuONPs extended to 22 days compared to bare CuONPs. In study reported by Tang et al. (2018) improved water resistance of alginate film was observed after addition of TiO_2_NPs and AuNPs by water contact angle test. Otherwise, the incorporation of cellulose nanofibers (CNFs) into glycerol plasticized starch-based polymer improved its thermal stability and reduced water vapor capacity. These resulted from high energy of chemical bonds formed between polymeric matrix and cellulose nanofibers as well as highly porous structure filler (CNFs), which increased the tortuosity of matrix and crystallinity of nanocomposite (Ahuja et al., 2021). Karimi Sani et al. (2021) reported that addition of zirconium oxide nanoparticles (ZrO_2_NPs) into potato starch/apple peel pectin-based films decreased its moisture content. The higher concentration of the nanoparticles the lower water vapor permeability of the films was observed. Moreover, these authors also modified composite films with microencapsulated *Zataria multiflora* essential oil that together with ZrO_2_NPs greatly increased the antioxidant properties, melting point and glass temperature of the film. The presence of essential oil reduced the crystal structure of the film while both components created porosity in the film structure. The optimum active films showed increased shelf life of the quail meat.

The development of polymer nanocomposites show more benefits and different mechanical, thermal, optical, and physico-chemical properties to pure polymers that can be used for variable applications (Duncan, 2011; Mathew and Radhakrishnan, 2019; Rahim et al., 2020) and also offers great functionality (Kausar, 2020; Karimi Sani et al., 2021).

## 3 Eco-friendly polymer-based delivery of agrochemicals: Minimal use with maximum efficacy

**FIGURE 3:**
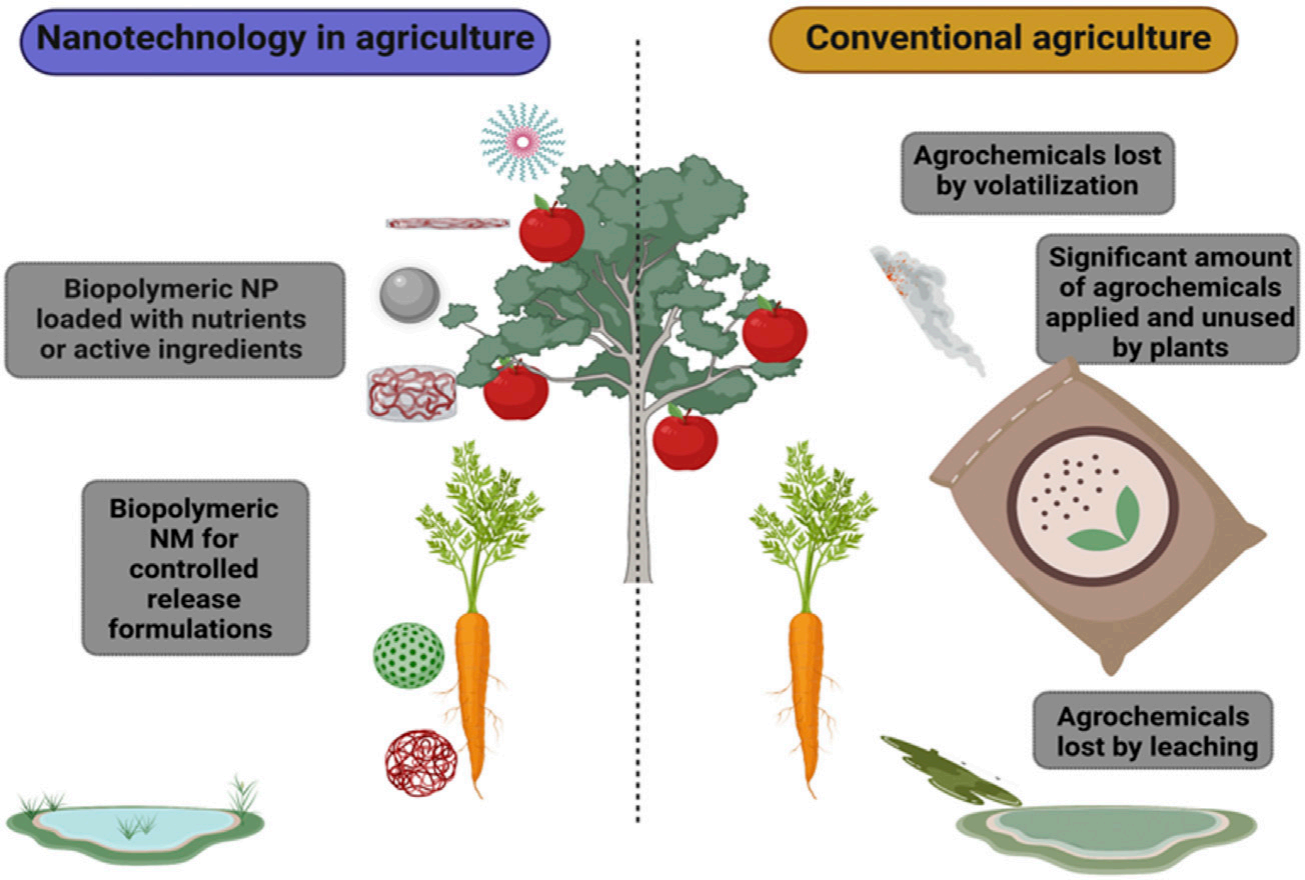
Advantages of nanotechnology in agriculture against conventional agriculture.

A current challenge in agriculture is to obtain more food production for a growing world population in less *per capita* land surface. For this, the use of fertilizers and other agrochemicals manages to increase the volume of agricultural products, however, they have negative environmental implications. For instance, soil nitrogen is lost by volatilization, this loss can reach 70%. Even a large proportion of the applied urea is lost by volatilization, leaching, or by incorporation into the soil (Avila-Quezada et al., 2022). Furthermore, some micronutrients are not available to plants (Madrid-Delgado et al., 2021) mainly in calcareous soil (Olivas-Tarango et al., 2021). For this reason, it is essential to have new nanotechnology-based fertilizers on the market. Moreover, pesticides are also largely lost from the field to other non-target sites due to weather factors (Materu et al., 2021) (Figure 3).

Nanomaterials (NM) are promising for use in agriculture because they have advantages, among the main ones is the low amount of product applied, remain for the desired time in the environment thus the active ingredients can be dosed and therefore control the pest or pathogen (Singh et al., 2020).

To increase the availability of nutrients for plants or the active ingredients against pathogens in sustainable agriculture, controlled release systems are the most viable option, for freeing themselves in a slower, sustained, and more directed way (Kamaly et al., 2016).

The nanomaterials designed for controlled release are the current trend in sustainable agriculture due to its long shelf life, in addition to the active ingredient is transferred by regulated permeation to a target site, for instance, nutrients are released as ions soluble in the soil (Shi et al., 2014).

The nanonutrients are delivered directly in an emulsion with the nanoelement, administration of the encapsulated nanoelement designed for slow controlled release or through complex nanocapsules incorporated in the matrix of an organic polymer that serves as a vehicle (de Olivera et al., 2019). The success of nanoencapsulated fertilizers and pesticides is the slow release and protection of the active ingredient with a hydrophilic coating to absorb water, swell, dissolve and release the active ingredients. The slow release is related to the stability of the coating nanocomposites (nanocapsules, nanospheres, micelles, and nanogels) (Sinha et al., 2019; Fertahi et al., 2021).

Biopolymers have agricultural applications because they are biodegradable, biocompatible, bioactive, and hydrophilic. Natural biopolymers of plant, animal and microbial origin are excellent options to replace synthetic agrochemicals. Moreover, biopolymers by their origin are innocuous, besides, a polymeric formulation of bioactive agents is relatively simple to achieve (Rahim et al., 2020). Polymeric nanoparticles allow bioactive substances to be encapsulated and protect them from degradation by external factors such as the weather (Joy et al., 2022). Agricultural polysaccharide hydrogels as carriers for controlled release nutrients, reduce the amount of fertilizer applied, as well as providing easy diffusion from the root to the entire plant (Fertahi et al., 2021).

Moreover, polysaccharide-based hydrogels can be incorporated with other synthetic polymers to produce valuable polysaccharide materials which serve as fertilizer carriers (Ghobashy, 2020). Moreover, the combination of biopolymer with SiO_2_, which is an inert material, has many nanotechnological applications, such as the administration and nanoencapsulation of active ingredients. Holed and porous SiO_2_ nanoparticles allow the loading of molecules of interest as active ingredients within the nanolayer (Torabi and Mohammadi, 2013).

Due to all these advantages offered by biopolymers for the delivery of agrochemicals, it is concluded that they currently offer maximum efficiency with minimal use.

Nanocapsules, nanospheres, micelles, nanogels, and nanofibers are the most common polymer-based nanomaterials delivering active ingredients in agriculture (Sun et al., 2020).

### 3.1 Biopolymer as nanocarrier of nanofertilizers and micronutrients

Biopolymers such as alginate, cellulose, chitin or chitosan, hemicellulose, lignin, polypeptides, and polyesters, used as nanocarriers to encapsulate nutrients and avoid dissolution and oxidation, are an eco-friendly option due to their natural origin and therefore biodegradable when compared to bulk synthetic fertilizers (Mishra et al., 2018).

Chitosan is the most accepted biopolymer due to its innocuous origin for use in agriculture. It is also an easy-to-manipulate matrix to program the adsorption and slow release of the target active ingredient (Pandey et al., 2018). Due to its natural origin, chitosan provides protection to plant cells, unlike other biopolymers that can have a harmful effect when in contact with the plant (Khairy et al., 2022).

The cover of nanofertilizers is designed to be porous for the slow release of the nutrient content (Kubavat et al., 2020). The time and dose of nutrient release will depend on the plant’s requirement (Lawrencia et al., 2021).

Among the most common nanofertilizers applied are urea, ammonium nitrate, ammonium sulfate, sulfuret, calcium nitrate, calcium phosphate, mono and diammonium phosphate, triple superphosphate, potash, silicon, zeolite, zinc, and so on (Rameshaiah et al., 2015; Rajonee et al., 2017; Alimohammadi et al., 2020; Carmona et al., 2022).

Agricultural waste products also have the potential to be used to manufacture nanofertilizers as banana peel, which is rich in minerals such as manganese, magnesium, and potassium. Banana peel-derived nanofertilizers studied by Hussein et al. (2019) contained chelated potassium, chelated iron, tryptophan, urea, amino acids, proteins and citric acid, and showed significant effects when tested for tomato and fenugreek seed germination (Hussein et al., 2019).

Commercial nanonutrients offer advantages such as controlled release due to the cover materials of the mentioned fertilizers. Controlled release refers to the slowly deliver of the nutrient over months (Vejan et al., 2021).

Some available products coated with patented biopolymers are Agrocote (United States), ESN Smart nitrogen (United States), Meister (Australia), Multicote (Israel), Nutricote of Florikan CRF (United States), Osmocote (United States), and zeolites have been generally used as fertilizers for fruit trees, coffee, bananas, sugar cane, vegetables, potatoes, rice, corn, and wheat, among others.

In addition, in the market, it is possible to find nanofertilizers such as Nano-Gro™ (United States), Nano-green (India), Nano-Ag Answer (United States), Biozar nanofertilizer (Iran), Nano max NPK with microelements and microorganisms (India), Master Nano chitosan organic fertilizer (Thailand), NanoMax (PowerMax) (Taiwan), TAG Nano fertilizer (India), Nanofertilizer LITHOVIT (Germany) and more. Their use is common in crops such as vegetables, fruit trees, wheat, rice, cotton, tea, and others.

Finally, biopolymers as nanocarriers of nanofertilizers or micronutrients have advantages over conventional fertilizers such as the low amount applied and the controlled release of the nutrient, which is more profitable to increase crop production and fruit quality (Elshamy et al., 2019; Lawrencia et al., 2021). Therefore, the future of nanofertilizers is promising because of the ecological approach.

### 3.2 Biopolymer as nanocarrier of fungicides/bactericides/viricides

Although other polymers can be applied in agriculture, chitosan is one of the most important enhancers of plant defences, as this biodegradable polysaccharide hydrogel forms protection barriers in plants and help the plant to develop defence responses against pathogens (Table 1). These properties of chitosan-based barriers have been tested with great efficacy against fungi and oomycetes (Vasyukova et al., 2000).

**TABLE 1:** Carrier systems and antimicrobials with the potential to be used as carriers for molecules or active ingredients for plant production.

Antimicrobial/carrier system	Effect	References
Chitosan	Control of plant pathogenic viruses: alfalfa mosaic virus (ALMV), tobacco necrosis virus (TNV), tobacco mosaic virus (TMV), peanut stunt virus (PSV), cucumber mosaic virus (CMV), and potato virus X (PVX)	Pospieszny et al. (1991)
Chitosan	Control of plant pathogenic virus: alfalfa mosaic virus, bean goldish mosaic virus, peanut stunt virus, tobacco necrosis virus, tobacco mosaic virus, potato virus X, potato virus Y, figwort mosaic virus, cucumber mosaic virus, bean yellow mosaic virus, bean common mosaic virus, potato spindle tuber viroid	Chirkov (2002)
Nano-chitosan	Control of *Ralstonia solanacearum*	Khairy et al. (2022)
Chitosan	Control of plant pathogenic fungi: *Fusarium oxysporum f. sp. radicis-lycopersici*	Palma-Guerrero et al. (2008)
Chitosan	Control of *Botrytis cinerea*	Badawy et al. (2005)
Chitosan	Control of *Pyricularia grisea*	Badawy et al. (2005)
Chitosan	Control of *Phytophthora infestans*	Vasyukova et al. (2001)
Nano-silica	Fertilizer	Singh and Endley (2020)
Nanoclay	Enhancer of plant growth	Nisar et al. (2017)
Nano-chitosan NPK fertilizer	Enhancer of growth and productivity of potato plant	Elshamy et al. (2019)
Nanozeolite	Enhancer of plant growth	Khati et al. (2018)

Moreover, chitosan can be used in combination with other materials (e.g., montmorillonite) to encapsulate nutrients or active ingredients of pesticides (dos Santos et al., 2015). Also, hydrogels with plant repellents (essential oils) are encapsulated in nanoparticles for plant protection (Oliveira et al., 2019; Sampathkumar et al., 2020). Some reports showed chitosan-based micelles as a controlled release formulation for biosafe pesticide delivery (Lao et al., 2010; Zhang et al., 2013; Xu et al., 2018; Feng et al., 2020).

### 3.3 Biopolymer as nanocarrier of insecticides

Polymer-based materials have been also found to be effective carriers for insecticides, mainly by increasing their solubility in water (Lao et al., 2010; Feng and Peng, 2012). The microspheres composed of chitosan and cashew tree gum were developed and loaded with essential oil of *Lippia sidoides* active against larvae of *Aedes aegypti* to use as a bioinsecticide to control larvae proliferation. This chitosan-based capsules showed prolonged larvicidal effect (Paula et al., 2010). Similarly, microcapsules of alginate and chitosan were found to be suitable matrice to carry nano-imidacloprid bioinsecticide. Interestingly, this carrier system allowed for up to eight times longer release of insecticide when compared with an insecticide used alone. Moreover, release time dependent on the concentration of alginate and chitosan used for encapsulation (Guan et al., 2008). The amphiphilic derivative of chitosan, N-(octadecanol-1-glycidyl ether)-O-sulfate chitosan was used to form spherical polymeric micelles (167–204 nm size) for encapsulation of insecticide. These nanoparticles were formed by self-assembly in aqueous solution and increased 1,300-fold solubility of rotenone in water providing its sustained release (Lao et al., 2010). The development of carboxymethyl chitosan nanoparticles with ricinoleic acid as an emulsifier for azadirachtin was found to be useful as an insecticide agent for agricultural applications due to the slow release of the active compound. These spherical particles in a size range of 200–500 nm showed good polydispersion, smooth high zeta potential, and solubilization in the water of the lipid-soluble azadirachtin (Feng and Peng, 2012).

## 4 Role of natural polymer-based hydrogels

The presence of water in the soil is a key factor for the growth and efficient production of crop plants and significantly affects agricultural sector during drought seasons or in regions with permanent water deficiency (Bogati and Walczak 2022). In agriculture, hydrogels and their composites are most commonly used as soil conditioners to facilitate the growth of crop plants by providing nutrients and water (Ghobashy 2020). Hydrogels are three-dimensional polymer networks of hydrophilic nature, that are not soluble in water, but effectively store and gradually release the water. They may be formed by using natural polymers of plant, microbial, algal, fungal or marine animal origin as well as hybridization with a synthetic one. Among them are those based on polysaccharides (chitosan, alginate, carrageenan, starch, cellulose, and pullulan), proteins (silk, keratin, and collagen) (Varghese et al., 2020; Rai et al., 2021b).

Hydrogels are used to improve the water ability of soils and reduce the drought stress of crop plants as they can adsorb water solutions up to a hundred times their weight (Guilherme et al., 2015; Hasan and Abdel-Raouf 2019). They play a major role in tissue regeneration, drug delivery, smart electronic devices, etc. There is considerable progress in using polysaccharides as a base for such composites (hydrogels), due to their eco-friendly nature, biodegradability, biocompatibility, bioactivity, and non-toxicity (Jiang et al., 2020).

Studies on biopolymer-based hydrogels showed superior water capacity, from 80 g g^−1^ water storage of cellulose-based hydrogel (Demitri et al., 2013) by 290 g g^−1^ water storage of chitosan-based hydrogel combined with aluminium chloride hexahydrate (Zhang et al., 2022) to 329 g g^−1^ water storage of cellulose/chitin hydrogel (Kono and Zakimi 2013). In addition, the great interest in hydrogels is due to their ability to retain water in the soil over the long term (Tomadoni et al., 2019). To obtain novel and innovative products with improved structural properties and functionality some solutions are proposed, including optimization of the synthesis process by incorporation of nanomaterials into natural polymer-based hydrogels (Sohail et al., 2022). For instance, the increased liquid absorption was achieved by incorporating zinc oxide nanoparticles (nZnO) into guar gum-pectin/polyacrylamide hydrogel composite (GG-PC/PAAm/ZnOx). It has been suggested that the improved swelling may be related to water uptake to compensate for the increasing ionic osmotic pressure resulting from the presence of nZnO and/or altered structure of pores and expanding the polymer network, subsequently increasing the space for water (Sayed et al., 2022).

The effectiveness of hydrogels depends on both their composition and the active ingredient embedded in the composite. Treatment of infected lettuce seedlings with chitosan-based hydrogel loaded with copper oxide nanoparticles (CuONPs) resulted in suppression of disease caused by *Fusarium oxysporum* f. sp. *lactucae.* Authors suggest that the mechanism of action involves direct interaction of Cu-based composite with fungal cells. In addition, seedlings under exposure to Cu-chitosan hydrogel were stimulated to increase nutrient uptake and improved regulation of metabolites production, including salicylic acid (SA), jasmonic acid (JA) and abscisic acid (ABA), further enhancing the plant’s immune response. The implementation of biodegradable and environmentally friendly hydrogels can reduce the dose of chemicals introduced into the environment and thus minimize adverse effects on non-target organisms, ecosystems, and human health (Shang et al., 2021). Agrochemical-loaded hydrogels are used for seed coating to improve seed germination and seedling growth, as well as their resistance to pathogens. Some studies have shown improved hydrogel properties by introducing micellar domains. Defined as self-organizing structures, micelles can be formed by low-molecular-weight surfactants as well as suitably modified polymers. It is proposed that they can be used to control the release rate of active compounds, increase solubility and bioavailability, and minimize chemical degradation (Pekař, 2015; Ye et al., 2015; Nascimento et al., 2020).

As heavy metal and other harmful soil contaminations have become a serious problem in agriculture another noteworthy issue is the potential use of hydrogels for soil remediation. Bio-absorbent properties were displayed by carboxylmethylcelullose (CMC) (Sekine et al., 2020), carboxymetylcellulose-starch-gelatin hydrogel (Devasia and John 2021), cellulose/lignin (Shan et al., 2021), and chitosan (Bhullar et al., 2022) which possess a high ability to the removal of diverse dyes and heavy metals. Therefore, nanohydrogel sheets (synthesized of guar gum and soya lecithin) successfully absorbed thiophanate methyl with maximum capacity 59.205 mg/g, suggesting their potential in the environment management for decontamination from fungicides (Sharma et al., 2018). Furthermore, it was found, that embedding lignin-based hydrogel with FeS nanoparticles resulted in an almost 6-fold increase in the sorption capacity of Cd to 61.77 ± 1.09 mg g^−1^. The composite is recyclable (by acid etching of Cd ions), thereby enabling its reuse (Liu et al., 2020). In turn, Kamel et al. (2020) prepared CMC-based hydrogel combined with magnetite NPs and porous carbon (PC) for lead-ions and methylene blue dye removal. Incorporation of Fe_3_O_4_ NPs and PC increased the sorption capacity and removal efficiency. These effects were associated with formation of highly porous structure and stronger attraction of contaminants.

There are several benefits when natural polymers (polysaccharides) are used in combination with synthetic hydrogel Ghobashy (2020), as given below.

(i) Enhancement of the gelling capacity of polysaccharides in water so that they swell
(ii) Sustained release of fertilizers depending on enzymatic degradation and crosslinking density
(iii) Resist the degradation of polysaccharides in natural conditions
(iv) Enhancement of binding affinity of ions present in the soil
(v) Demonstrate resistance to UV light and chemicals
(vi) Increase the thermostability of natural polymers.

## 5 Bioactive nanocoating for smart nanoactive packaging

**FIGURE 4:**
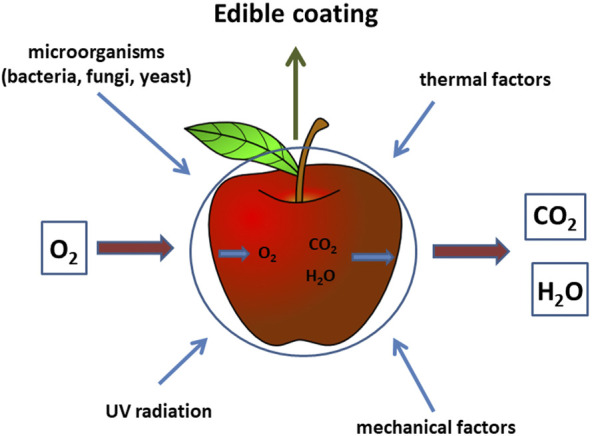
Role of nanocoatings in food production.

Food can be packaged according to three levels, such as primary, secondary and tertiary packaging. A coating or foil that directly surrounds the food and has contact with it and thus affects the quality of the product is included in the primary packaging. Secondary packaging already covers products previously covered by primary packaging, and tertiary packaging is called outer packaging, which is very often used for transport or distribution (Ahari and Soufiani, 2021). Edible coatings are thin and transparent layers (films) applied to the surface, mainly fruits, that are made of materials intended to come into contact with food (Figure 4), and added to or as a substitute for the waxes naturally present on the surface of the fruits (Mahela et al., 2020).

Nanocoatings are ultra-thin layers in the range of 1–100 nm thick applied on the substrate without altering the surface of the substrate and act as a barrier to different gases and food-deteriorating microbes (https://www.nanowerk.com/nanotechnology-news/newsid=47370.php), as shown in Figure 4.

Polymers are excellent agents for food packaging owing to their barrier properties to keep products away from food deteriorating microbes, retain their quality (freshness), and enhance their shelf-life. Each polymer has a different property. Some polymers are barriers to oxygen, while others may be barriers to water vapor. These are influenced by several factors including polarity, crystallinity, hydrogen bonding, etc. (Sarfraz et al., 2020). The barrier properties of polymers can be enhanced by using nanofillers leading to the formation of nanocomposites, as mentioned previously.

**FIGURE 5:**
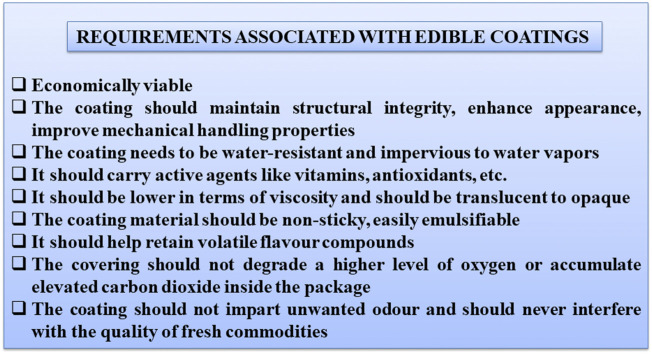
Requirements for edible coatings in food industry.

Overall, the coatings to be used in food industry must meet several important criteria, as shown in Figure 5.

However, processed and fresh fruits have specific packaging requirements. Fruit films and coating, as a part of the product, are fully edible therefore the components from which they are produced must be non-toxic, and meet all safety standards (i.e., their composition should be GRAS—Generally Recognized as Safe). They also should not alter the taste of the product (Burdock and Carabin, 2004; Yousuf et al., 2018). Moreover, the fruit coating should have low water vapor permeability to delay drying out and limits the permeability of oxygen and carbon dioxide to slow down respiration and metabolic activity as well as the maturation process (Sharma A K et al., 2019). The edible coatings/films made of various types of biopolymers combined with various additives such as glycerol, aloe resins, polyphenols or urea are less than .3 mm thick (Embuscado and Huber, 2009; Castro-Muñoz and González-Valdez, 2019; Morales-Jiménez et al., 2020). The components of coatings are well dissolved and dispersed in water, alcohol, the mix of water and alcohol or other solvents prior to application to the product using dipping, spraying, brushing or panning methods, followed by drying (Bourtoom, 2008; Díaz-Montes et al., 2021). These bioactive polymers when possessing antimicrobial, antioxidant, water and oxygen barriers/scavengers extend the shelf life of coated products (Malhotra et al., 2015). The edible coating or film is mainly selected on the basis of water solubility, hydrophilicity and hydrophobicity nature, ease in the formation of coatings and sensory properties to convey information about food quality and increase the safety of foods (Sharma A et al., 2019; Pirsa et al., 2022).

### 5.1 Lipid-based coatings

Waxes such as carnauba wax, beeswax, paraffin wax and resin are neutral lipids used to coat fresh fruit and vegetables (citrus, apple, mature green tomato, cucumber, asparagus, beans, carrots, eggplant, and turnip) to improve the appearance of products and protect them against moisture (Sharma A et al., 2019). The lipid-based coatings are compatible with other coatings used and have high water vapour and gas barrier properties (Sharma A et al., 2019).

### 5.2 Polysaccharide based coatings

Polysaccharides such as starch, pectin, carrageen, alginate, gill, gum, chitosan, cellulose and its derivatives often can use as stabilizers, thickening substances, gelling agents, and strengthening due to their high viscosity (Stephen and Phillips, 2016; Eghbaljoo et al., 2022). Polysaccharide-based coating has shown storage properties of gases, favored compounds, and fatty substances (Dhanapal et al., 2012; Cazón et al., 2017).

Starch-based films show clarity, elasticity, tasteless, non-toxic, odorless and good gas barrier properties (Chiumareli and Hubinger, 2012; Pelissari et al., 2019) while dextrin (a derivative of starch) films have good water vapor resistance (Sharma A K et al., 2019).

Pullulan-based coatings are transparent, edible (GRAS), tasteless and inert to food ingredients, show non-toxic effect, glue properties, high mechanical strength and restricted permeability to oxygen and carbon dioxide gases (Wu and Chen, 2013; Kraśniewska et al., 2017). These coatings are used for coating apples, blueberries, strawberries, kiwi fruit, carrots, peppers, and also Brussels sprouts (Kraśniewska et al., 2017; Ganduri, 2020). Overall, pullulan, a carbohydrate produced by *Aureobasidium* spp and used as an effective oxygen barrier coating for fruits and vegetables restrict growth of aerobic microorganisms, including molds and bacteria, that are mainly responsible for food spoilage and thus can extend food shelf life. Interestingly, this effect was achieved without additives of any antimicrobial agents into the polymer (Chlebowska-Śmigiel and Gniewosz, 2009). However, it was suggested that pullulan as a hard-to-absorb carbon source for bacteria and fungi can also itself restrict microbial development on the surface of food and therefore extends its shelf life (Kraśniewska et al., 2017). Moreover, pullulan can be utilized in food packaging in combination with essential oils and other agents of antimicrobial and antioxidant activities. However, there are some limitations of pullulan use in food packaging, such as the high cost involved in the production of this polymer (Singh and Saini, 2014).

Cellulose derivatives such as carboxylmethylcelullose (CMC), methylcellulose (MC), hydroxypropyl cellulose (HPC) and hydroxypropyl methylcellulose (HPMC) are most often used in the food industry. Cellulose derivatives are water soluble, non-ionic and compatible with surfactants (Sharma P et al., 2019).

Chitosan films are stable and show mechanical and barrier properties. They are used as antimicrobial coating for strawberries, cucumbers and bell peppers, and as a gas barrier for apples, pears, peaches and plums (Bourtoom, 2008).

### 5.3 Protein films

Proteins such as gelatin, casein, corn zein, wheat gluten, and mung bean, peanut soy and whey proteins are commonly used in forming edible films/coating (Bourtoom, 2008). Proteins film exhibits good gas and lipid barrier properties (Popović et al., 2012), especially at low relative humidity (Šuput et al., 2015).

**FIGURE 6:**
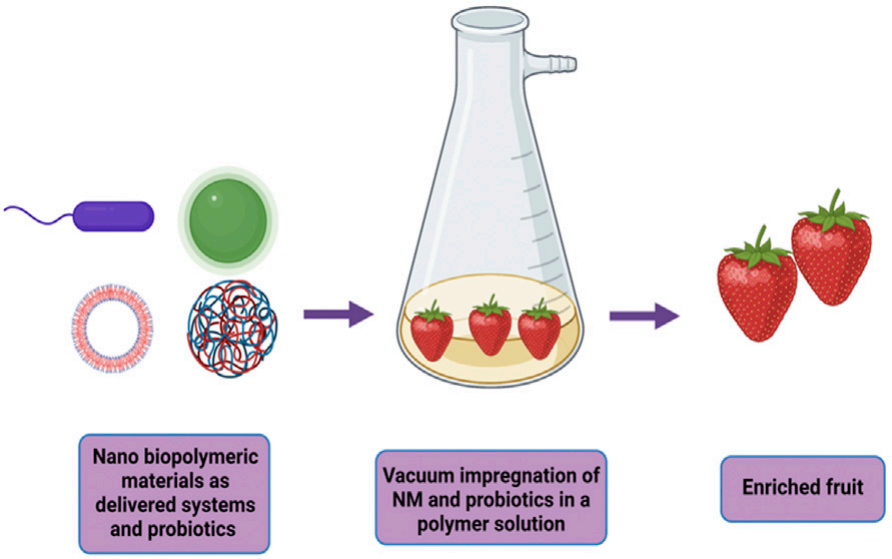
Vacuum impregnation of nano-delivery systems and probiotics with atmospheric pressure.

Edible coatings or films on fresh vegetables and fruits are used to block oxygen, microorganisms, and moisture, and as protective and preservative barriers against sunlight damage (Sharma S et al., 2019; Tahir et al., 2019), as shown in Figure 4. Interestingly, the vacuum impregnation experiment of probiotics in fruit developed by Soto-Caballero et al. (2021) could be used in industry to form polymer-based edible nano-coatings with nano-encapsulated probiotics to obtain enriched fruits, as proposed in Figure 6.

The efficiency of the coatings depends of their properties, as gas, water and lipid permeability, and odor, but these, in turn, depend strictly on the chemical composition and structure of polymers, product characteristics and storage conditions (Skurtys et al., 2014). One of the most important properties of nanocoatings is the mechanical properties. The durability of the coatings is related to the ability of the polymers to create molecular bonds between polymer chains and the system of polar groups as well as the polarity of polymer chains contribute to increasing ionic interactions between the chains (Gontard and Guilbert, 1994; Hammam, 2019). Skurtys et al. (2014)) reported that protein films exhibit lower tensile strength than polysaccharide films. However, protein-based nanocoatings have been found to possess excellent barrier properties (Chen et al., 2019). These coatings have an oxygen barrier with an oxygen permeability of 260, 500, 540, and 670 times less than methylcellulose, polyethylene, starch and pectin, respectively. Moreover, Rukmanikrishnan et al. (2020) prepared K-carrageenan/lignin film with good thermal and mechanical properties and 100% UV protection while water vapor barrier of this polymer material was limited.

Food during production, storage or transport may be contaminated with microorganisms that leads to spoilage of products (He and Hwang, 2016). Therefore, packaging films with antimicrobial activity are highly desired in the food industry. As far as active packaging is concerned, there are some bioactive agents including antimicrobials, enzymes, and antioxidants used as a component of polymer-based packaging that can destroy the microbes (bacteria, fungi, protozoans, etc.) or inhibit their entry and therefore this area of research is increasing the attention of the scientific community (Khezerlou et al., 2018). In this context, nanocomposites play a pivotal role due to their barrier and antimicrobial properties (Basavegowda and Baek 2021). Due to the application of antimicrobial agents and nanoparticles such as AgNPs, CuNPs, etc. this type of packaging is known as “active packaging.” With the high surface-area-to-volume ratio and antipathogenic nature, nanocomposites are suitable candidates for active food packaging. To date, the nanomaterials that are used in antimicrobial food packaging include AgNPs, CuNPs, AuNPs, ZnONPs, TiO_2_NPs, MgONPs, nanoclays (montmorillonite; MMT), natural antimicrobials such as essential oils, sesquiterpenes, nisin, thymol, isothiocyanate, carvacrol, nisin, vanillin, cinnamon, bacteriocins (capidermicin, carnobacteriocin, duramycin, enterocin, mutacin, etc.), and synthetic antimicrobials (Table 2).

**TABLE 2:** Activity of nanomaterials/nanocomposites used for food packaging.

Active component	Polymer matrix	Biological activity	Concentration of active compounds	Type of food	References
Silver–copper nanoparticles	Fish skin gelatin	Antibacterial: *L. monocytogenes, S. typhimurium*	2% (w/w) NPs	—	Arfat et al. (2017a)
Silver nanoparticles copper nanoparticles	Agar	Antibacterial: *L. monocytogenes, S. typhimurium*	.5% (15 mg), 1% (30 mg), 2% (60 mg) and 4% (120 mg)	—	Arfat et al. (2017b)
Silver–copper nanoparticles	Gaur gum	Antibacterial: *L. monocytogenes, S. typhimurium*	.5%–2% NPs	—	Arfat et al. (2017c)
Cinnamon essential oil and TiO_2_	Sago starch	Antibacterial: *E. coli*, *S. typhimurium*, *S. aureus*	0%, 1%, 3%, and 5%, w/w) of TiO_2_ and CEO (0%, 1%, 2%, and 3%, v/w)	Fresh pistachio	Arezoo et al. (2020)
Tarragon essential oil	Chitosan/gelatin	Antioxidant	chitosan to TEO (1:0, 1:0.2, 1:0.4, 1:0.6, 1:0.8 and 1:1)	Pork slices	Zhang et al. (2014)
ZnO	Alginate	Antibacterial: *S. typhimurium, S. aureus*	N/A	Ready-to-eat poultry meat	Akbar and Anal 2014
TiO_2_	Chitosan	Antibacterial: *S. aureus*, *E. coli*, *S. typhimurium*, *P. aeruginosa*	Different TiO_2_ concentrations (0, .25, .5, 1% and 2% w/w)	Aimed for postharvest applications of fresh produce	Siripatrawan and Kaewklin (2018)
Blackberry powder	Arrowroot starch	Antioxidant	Sprinkling with 0, 20, 30, and 40% (blackberry solids mass/biopolymer mass) blackberry particles	—	Nogueira et al. (2019)
Coconut water	Coconut protein precipitate	Antioxidant	N/A	—	Rodsamran and Sothornvit (2018)
Oregano essential oil	Citrus peel pectin	Antibacterial: *E. coli*, *S. aureus*, *L. monocytogenes*	.24 mg/mL	Shrimp and cucumber slices	Alvarez et al. (2014)
Clove, fennel, cypress, lavender, thyme, herb-of-the-cross, pine and rosemary essential oils	Chitosan and gelatin	Antibacterial: *P. fluorescens, S. putrefaciens, P. phosphoreum, L. innocua, E. coli, L. acidophilus*	Food grade clove essential oil was incorporated in a proportion of .75 mL/g biopolymer	Fish preservation	Gómez-Estaca et al. (2010)
Clove essential oil	Soy protein isolate and microfibrillated cellulose	Antioxidant	N/A	—	Ortiz et al. (2018)
Extracted spent coffee ground	Cassava starch	Antioxidant	50 g/500 mL	—	Ounkaew et al. (2018)
Cinnamon oil	Soybean polysaccharide	Antioxidant, Antibacterial: *S. aureus* and *S. pyogenes*	.6% and .8% concentration of cinnamon oil	Meat products	Ghani et al. (2018)
Curcumin	Chitin nanofiber	Antioxidant	1 mg/mL, 2.5 mg/mL and 5 mg/mL	—	Yang et al. (2020)
Cinnamon oil	Chitosan-whey protein/zein	Antibacterial: *E. coli, S. aureus*	2% and 4% (w/w) amounts	—	Vahedikia et al. (2019)
AgNPs	Cellulose nanofibril	Antibacterial: *E. coli, L. monocytogenes*	0, 1, 2.5, 5, and 10 mg/mL	—	Yu et al. (2019)
Orange-peel oil	Corn starch	Antioxidant	OPO and corn starch (3:10, w/w)	—	Wang et al. (2019)
ZnO	Bovine Gelatine	Antifungal: yeast	5% (based on dry gelatin)	Sponge cakes	Sahraee et al. (2020)
ZnONP	Chitosan	Antibacterial: *E. coli*	2% (w/v) ZnO nanoparticles	White brined cheese	Al-Nabulsi et al. (2020)
Garlic extracts	Biodegradable starch	Antibacterial: *Salmonella* sp., *S. aureus*	NA	Milk products, fatty foods, liquid, Acidic and dry foods	Baysal and Dogan (2020)
Metal ions (silver, copper) and metal oxides nanoparticles (ZnO, TiO_2_)	Fish gelatin	Barrier properties	ZnONPs (3% (w/w); TiO_2_ (.5–2 g, w/w)	—	Hosseini and Gomez-Guillen (2018)
K-carrageenan	Cellulose nanocrystals	Mechanical properties, Barrier properties (water, UV)	9–7 wt%	—	Yadav and Chiu (2019)
AgNPs	Cellulose	Antibacterial: *B. stearothermophilus*	5%, 10% and 20% w/w concentrations	—	Vivekanandhan et al. (2012)
ZnONPs, AgNPs	Chitosan	Antibacterial: *E. coli, S. typhimurium, S. aureus, B. aureus, L. monocytogenes*	Ag-NPs (.021–.120 mg); ZnO-NPs (.01 mg)	—	Youssef et al. (2015)
AgNPs-TiO_2_NPs	Chitosan	Antibacterial: *E. coli*	.38 μg/mL	Fruits	Lin et al. (2015)
Silver	Chitosan	Antibacterial: *B. subtilis, E. coli* Antioxidant			Nandana et al. (2022)
AgNPs-corn extract	Chitosan	Antibacterial: *E. coli, S. aureus, Salmonella* sp.*, L. monocytogenes* Antioxidant	25 and 50 μg/mL	—	Qin et al. (2019)
AgNPs	Pullulan	Antifungal: *A. niger*	.628–1.710 mg/mL	—	Pinto et al. (2013)
Lysozyme nanofibers	Pullulan	Antibacterial: *S. aureus* (lysozyme resistant strain) Antioxidant	15.0 wt%	—	Silva et al. (2018)
AgNPs	Pullulan, Pectin	Antibacterial: *L. monocytogenes, S. typhimurium, S. aureus, B. cereus*	N/A	—	Lee et al. (2019)
Ag and ZnO	Cassava starch/Agar	Antibacterial: *P. aeruginosa, S. aureu*s	Different concentrations (.5, 1, 1.5 and 2 mM) of Ag nanoparticles and ZnO nanoparticles	—	Mahuwala et al. (2020)
Cardamom extract, CeO2 nanoparticles	Pectin	Antibacterial: *E. coli, S. aureus*	Cardamom extract (0, 3.75 and 7.5 mL/g); CeO2 nanoparticle (0, 2.5 and 5 mg/g	—	Karimi Sani and Alizadeh (2021)
Copper sulfide nanoparticle (CuSNP), Nigella sativa essential oil	Fish skin gelatin and chickpea protein isolated	Antibacterial: *E. coli, S. aureus*	.25 and .5% copper sulfide nanoparticle (CuSNP), *Nigella sativa* essential oil (.015% and .03%, w/w of protein)	—	Rasul et al. (2022)

The antimicrobial activity of biopolymer-based nanocoatings against bacteria and fungi has been shown by many authors, as seen in Table 2. Moreover, Tabassum and Khan (2020) evaluated the freshness of papaya (gas exchange, and sensory quality) coated with alginate-based edible polymer with thyme and oregano essential oils for 12 days. Although the content of essential oils extended the shelf life of papaya, the sensory performance decreased to an unacceptable level.

The antioxidant properties of biopolymer-based nanocoatings containing orange peel oil or curcumin have been studied by Yang et al. (2020) and Zhang et al. (2020). Topuz and Uyar. (2020) and Aziz and Karboune. (2018) reported that natural active agents such as ascorbic acid, phenols and polyphenols, ferulic acid, a-tocopherol and also phycocyanin are very good antioxidant agents. Undoubtedly, oxidative processes such as the oxidation of carotenoids, chlorophyll, anthocyanins and degradation of vitamins occurring in stored or transported food products cause rancidity and loss of color of the products (Vasile, 2018). Freshly cut fruits rapidly browning as a result of the conversion of phenolic compounds into dark color pigments in the presence of O_2_. Therefore, additives of antioxidants to an edible coating are a good way to deal with such an undesirable effect (Rojas-Grau et al., 2009).

## 6 Nano-polymer with risks of migration into food

As far as the risks related to the application of nanocomposites are concerned, it is important to assess, manage and govern risks meticulously (Iavicoli et al., 2017). The proper assessment of the effect of shape, size, surface charge, concentration, etc. of engineered nanoparticles used to form nanocomposites is a matter of great concern and should be studied carefully, and the risk management should be managed and regulated (Amini et al., 2014; Evans et al., 2017).

**FIGURE 7:**
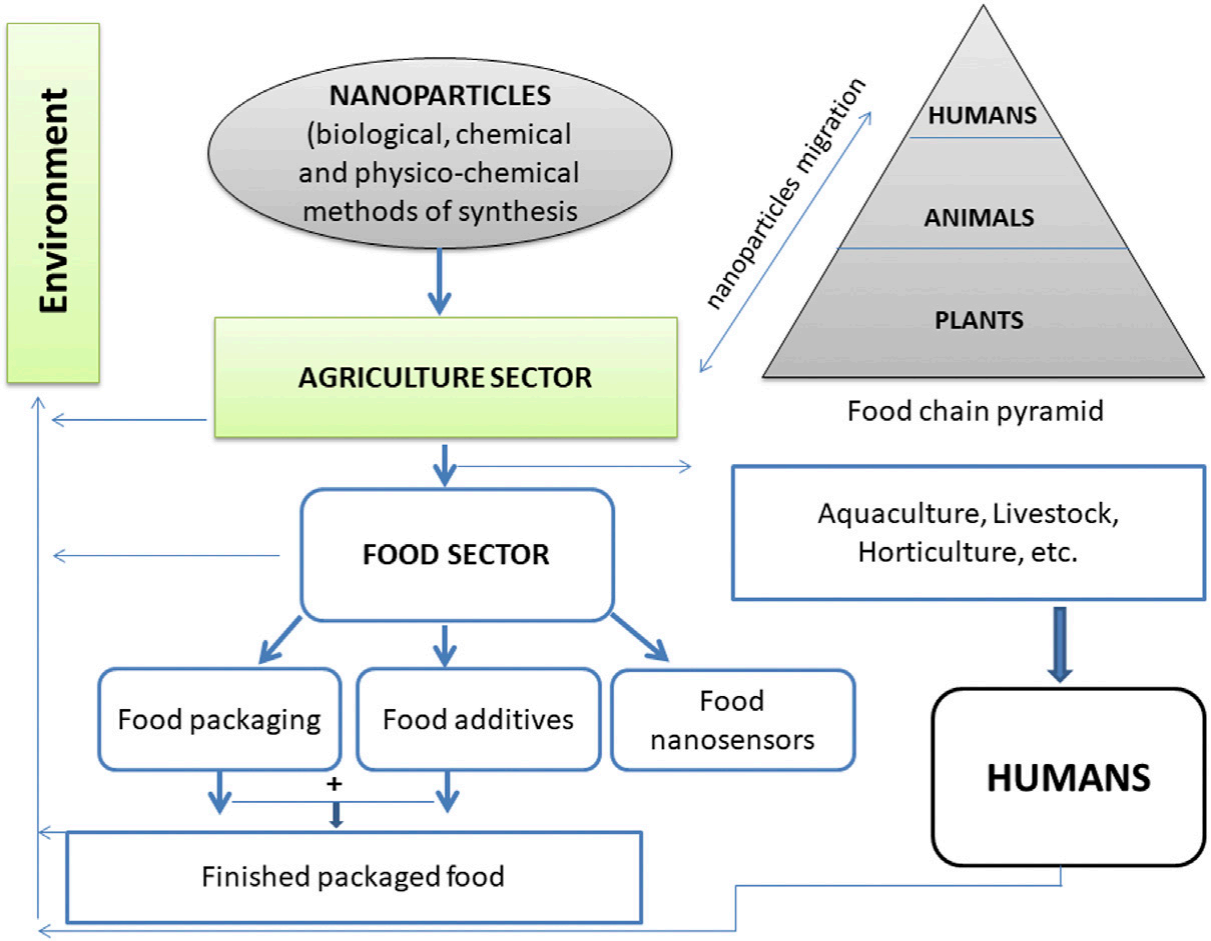
A proposed typical cycle of migration of nanoparticles from their source of synthesis to the agriculture sector, and then finally accumulate inside the human body through the finished packaged food.

Although nanotechnology has multiple applications in different fields, our understanding of toxicity still needs the support of more thorough studies to know the real picture of noxiousness. In this context, the use of polymer-based nanocomposites is a risk as nanoparticles may enter food and cause toxicity and allergy. This is possible due to the migration of packaging materials into food (Figure 7). Therefore, the migration test of nanomaterials is essential under controlled conditions (Honarvar et al., 2016). The migration of nanomaterials from polymer nanocomposites to food depends on the physicochemical properties of both nanomaterials and food. These include, but are not limited to shape, size, concentration, solubility, diffusion of nanomaterials and type of food, pH, and duration of interaction with the nanomaterials used in packaging (Huang et al., 2015). Furthermore, the authors raised the following questions, that need to be addressed concerning the toxicity due to the migration of nanomaterials, about physicochemical characterization of nanomaterials, protocols to assess the migration of nanomaterials, sophisticated methods to detect and characterize nanomaterials, how nanoparticles’ physical nature (shape, size, surface charge, etc.) is related to toxicity, and toxicokinetics after consumption.

Amini et al. (2014) cautioned that the toxicity caused by nanomaterials may be a disaster. Therefore, characteristics of the nanomaterials such as shape, size, solubility, and agglomeration should be studied in depth in order to understand the real problem of nanotoxicity. Similarly, Evans et al. (2017), have critically reviewed the genotoxic effect of nanomaterials that may affect cellular mechanisms, including DNA damage and cell division. On the one hand, the attractive and unique characteristics of nanoparticles make them important to use in various fields, but on the other hand, they are a potential threat to the environment and humans (Onyeaka et al., 2022).

However, there are some supporting studies that showed that there is no migration of nanomaterials into food if encapsulated properly in the polymer used (Bott and Franz, 2018).

There are regulations framed by the United States Food Drug and Administration (U.S. F.D.A.) and the European Commission (EU) which deal with the migration of nanomaterials into the food product that regulates (Paidari et al., 2021), as exemplified by EU directive No. 85/572/EEC. According to this directive the migration of nanomaterial should surpass the limit of 10 mg/dm^2^ (Hannon et al., 2015).

## 7 Challenges, chances, and consumers’ perception

Although agrifood nanotechnology has tremendous potential to face the global challenges of food security and sustainable crop production, there are some challenges that need to be overcome. The fate of the nanoparticles, bioavailability, ecotoxicity, etc., are the major issues that need to be addressed meticulously and convincingly (Ashraf et al., 2021). The nanomaterials used in polymer nanocomposites should be evaluated for their toxicity to soil microbes, and interaction with plants, environment, and animal models. Lowry et al. (2019) have discussed the opportunities and promises of nanobiotechnology, the challenges, and the need for a systems approach to design sustainable nanotechnology. Mishra et al. (2017) suggested overcoming the toxicity, optimizing the dose of nanoparticles, determining permissible limits, and designing experiments in the natural environment. Furthermore, the authors recommended the commercial application of biosynthesized nanoparticles in agriculture and food sectors.

Consumers’ role in accepting any technology depends on the safety of the technology used. In the present digitization era, people are more concerned about the food products that are promoting their health, but they are equally interested in the technologies which do not cause any harm to health and environment (Wansink and Chandon, 2014). The regulations and their proper implementation are essential for consumers. The engineered nanomaterials used in the products or nanocomposites should be addressed clearly for public acceptance. Recently, Siddiqui et al. (2022) reviewed the social and psychological factors of the consumers’ perception of non-packaging for food and food products. Among the social factors social concerns, norms and media play important role in attracting new nanocomposite-based technology. In addition to the social factors, there are psychological aspects such as awareness, motivation, attitude, beliefs, fear, and inherent habits which drive consumers to use new technology (Siddiqui et al., 2022). It is suggested that there is a need for the generation of awareness, attitude, and motivation among consumers to accept the use of new technology in food and also for sustainable agriculture (Siddiqui et al., 2022).

The use of nanopackaging and nanocomposites is increasing fast, but unfortunately, the efforts on framing and implementation of corresponding regulations have not been made. Usually, the regulations framed should emphasize food, health, and environment (Siddiqui et al., 2022).

Biopolymer-based formulation shows great potential in food preservation or crop plant protection. They reduce pathogens and contaminants in fresh and processed foods during storage and transportation, as well as exhibit biostimulant effects, reduce seedling damage and burn defects, improve the physical and chemical properties of soil, and increase water retention (Fertahi et al., 2021). However, before their practical application, there are some challenges to overcome such as the precise release of active ingredients in a controlled manner and the long-term stability of bioproducts. Furthermore, it is essential to deliver of appropriate concentration of nutrients or fertilizers at the relevant stage of plant growth. Biopolymer efficiency may be negatively affected by soil parameters and microbial activity (Spiridon et al., 2008; Weng et al., 2013; Ramili, 2019). Irfan et al. (2020) used a machine learning model to predict the kinetic of nitrate release from urea coated with starch-polyvinyl alcohol-cross-linked biopolymer considering many factors such as the method of synthesis and product properties. Estimated results were similar to release profiles from the experiments, making it a potential tool for predicting of nutrient release profiles from various biopolymer-based fertilizers.

The commercialization of biopolymers or their composites supplemented with nanomaterials has been relatively slow despite numerous studies. Research efforts should include scale-up studies to facilitate the transfer of products from the laboratory to safe industrial applications (Souza et al., 2020; Binod et al., 2021). Therefore, polylactic acid (PLA) films are commonly used for packaging fresh food (fruits, vegetables, fish, meats, and grains). Additional antimicrobial substances (peptides, amino acids, and essential oils) are loaded into PLA films to inhibit bacterial corrosion of food (Roy and Rhim, 2020; Subbuvel and Kavan 2022). For instance, polylactic acid and magnesium oxide (MgO) has been used to prepare large-scale biopolymer films for food packaging applications. The production process was transferred from a twin-screw extruder (5% PLA/MgO) to industrial-scale blown film extrusion (3% PLA/MgO), resulting in increased production from 45 g to 3 kg of each film, as well as improved mechanical, barrier, antibacterial, thermal and optical properties (Swaroop and Shukla, 2018; Swaroop and Shukla, 2019).

With increasing consumer awareness of environmental issues, the requirements for sustainable packaging as well as the use of biodegradable products to deliver plant protection and growth promotion supplies are becoming a new trend in the food and agriculture sectors (Yan et al., 2022). However, there are emerging concerns about the safe use of agrochemicals and food packaging materials with nanoadditives due to the risk of their leaching into food products (Sohal et al., 2018; Taherimehr et al., 2021). Nevertheless, there is still a need for further research on nano-biocomposites to address their impact on human health, as well as environmental issues, including studies of transport, cytotoxicity and ecotoxicity before introducing these materials into widespread use (Mohanty and Swain, 2017; McClements 2021). Vijayakumar et al. (2021) synthesized silver nanoparticles using cellulose biopolymer as stabilizing agent (Ce-AgNPs) and evaluated its cytotoxicity and phytotoxicity for potential biomedicine and agriculture applications. The results showed a dose-dependent cytotoxic effect against normal human keratinocyte (HaCaT) and human breast cancer (MCF-7) cells and similar IC50 values at level 5 and 4.9 μg/mL, respectively. Moreover, Ce-AgNPs decreased the germination and shoot length of wheat (*Triticum aestivum*) seedlings, whereas there was no adverse effect on the germination and growth of mung (*Vigna radiata*) seedlings. Thus, it is essential for the application of products incorporating nanomaterials to be rigorously tested for the occurrence of potentially undesirable effects on plants, animals, and humans (Sohal et al., 2018).

## 8 Conclusion

The emergence of nanotechnology has transformed different fields including food and agriculture. With various applications in agriculture for sustainable crop production and also to feed the ever-increasing population, nanotechnology has come to rescue mankind. In the present scenario, there is a high demand for use of biopolymers or natural polymers that are biodegradable without causing any hazard to the environment and living organisms. The application of nanomaterials impregnated or encapsulated in polymers like chitosan, pullulans, alginate and CMC, etc. for the delivery of agri-chemicals such as fungicides, insecticides, nanofertilizers, and micronutrients play a key role in efficient and slow delivery of chemicals. Such chemicals are used in minimum quantity but they are utlilized fully without the loss in drainage and thus not mixing into aquatic ecosystems. In natural environment, hydrogels are very important and facilitate to improvement of the water ability of soils and reduce the drought stress of crop plants as they retain moisture for a long duration by adsorbing water. These water hydrogels not only retain water but are also used for the slow delivery of fertilizers and micronutrients. Moreover, ultrathin-nanocoatings can be applied on the substrate without altering it to act as a barrier for different gases and food-deteriorating microbes. These nanocoatings (lipid, protein or polysaccharide-based) or encapsulation also augments antioxidant, and barrier properties, and thus nanocoatings on food packaging enhance the chance of increasing the shelf life of the food and food products. The barrier property of the nanocoatings can be further enhanced by organic and inorganic nanofillers. Although the application of nanomaterials coupled with biopolymers in food and agriculture applications is beneficial, it is argued by the scientific community that there are high chances of migration of the nanomaterials into food, which warrants further extensive research for food safety and security. Food toxicity is a matter of great concern, and therefore, a thorough assessment of toxicity risks, its management, and regulations governing such toxicity should be studied. The physical characteristics of the nanomaterials such as shape, size, solubility, and agglomeration, and their bioactivity need to be addressed to understand the toxicity of such materials. However, application of natural or biopolymers and their composites may provide a safer use of nanomaterials in food and agriculture. Furthermore, on one hand, there are a plethora of possibilities for application of polymer-based nanocomposites, while on the other hand, the fate of nanomaterials, solubility, and ecotoxicity are major challenges that warrant a deeper understanding of their applications. In application of any novel technology, public perception plays a key role. If they are not aware of the benefits and limitations of new technology, the efforts to commercialize the technology for the end users will be futile. However, this problem can be solved by arranging public meetings to generate awareness, attitudes, and skills among the common people.
